# Pseudoaneurysm of the Superficial Femoral Artery after Knee Arthroscopy

**DOI:** 10.7759/cureus.7559

**Published:** 2020-04-06

**Authors:** Patrick Carroll, Greg Fulton, Declan Reidy

**Affiliations:** 1 Trauma & Orthopaedic Surgery, Cork University Hospital, Cork, IRL; 2 Vascular Surgery, Cork University Hospital, Cork, IRL

**Keywords:** arthroscopy, orthopaedics, knee, vascular, pseudoaneurysm, stenting

## Abstract

An 84 year old gentleman underwent knee arthroscopy. He had x-ray proven left knee osteoarthritis. He had a number of medical co-morbidities including being on an anticoagulant for atrial fibrillation. He did not want a knee replacement.

A knee arthroscopy was performed which confirmed severe left knee osteoarthritis. Debridement of degenerative meniscus was performed. Three weeks post-operatively, the patient presented to the emergency department complaining of swelling and pain in his lower limb. He underwent an ultrasound venogram to look for a deep venous thrombosis (DVT). He was diagnosed with a pseudoaneurysm (PSA) of the superficial femoral artery (SFA). Subsequently, he was referred to the vascular surgery service who treated the PSA with covered stenting. The thigh and knee pain dissipated almost immediately.

We propose that this is the first PSA of the left SFA to be documented after a knee arthroscopy. The authors would like to acknowledge that knee arthroscopy for severe osteoarthritis is rarely performed in an octogenarian. However, as this patient had declined a total knee replacement (TKR) and injections were no longer providing him relief, knee arthroscopy was performed.

## Introduction

An 84 year old gentleman attended our Orthopaedic Surgery outpatient clinic due to left knee pain. He was subsequently diagnosed with x-ray proven severe left knee osteoarthritis. The patient has a background history of Hodgkin’s Lymphoma, atrial fibrillation, transient ischaemic attack, hypertension and cataract surgery. Of note, he was taking an oral anti-coagulant, Apixaban, and has had four cycles of chemotherapy.

The patient's knee pain affected his activities of daily living and shared decision making took place in the outpatient clinic. After considering the risks and benefits of a total knee replacement (TKR), the patient opted for non-operative management of his knee osteoarthritis. A standard non-operative management protocol was followed [1]. This included non-steroidal anti-inflammatory drugs, physiotherapy and intra-articular injections of local anaesthetic and steroid. 

Unfortunately, the patient had minimal relief from non-operative management and suffered from pain on a daily and nightly basis. He had difficulties with activities of daily living and requested another form of treatment other than a TKR. 

A discussion took place of the merits of knee arthroscopy for this patient. Knee arthroscopy in older aged patients is controversial and although there can be a small temporary improvement in their symptoms in the short-term, it is not recommended as this is of modest probability and uncertain [2]. The potential serious risks were explained to the patient including no improvement in pain, worsening of pain, knee stiffness, infection, artery or vein injury including a limb threatening injury, nerve injury, DVT, myocardial infarction, pulmonary embolism, a respiratory tract infection and death. After obtaining informed consent, the patient decided to undergo knee arthroscopy with the hope of a short-term benefit in symptoms.

## Case presentation

A left knee arthroscopy was performed on an 84 year old gentleman with severe knee osteoarthritis. The patient has a background history of Hodgkin’s Lymphoma, atrial fibrillation, transient ischaemic attack, hypertension and cataract surgery. Of note the patient takes an oral anticoagulant, Apixaban, for atrial fibrillation and has had 4 cycles of chemotherapy for Hodgkin's lymphoma. 

The patient presented to the emergency department 3 weeks after the arthroscopy due to left limb swelling and pain. The patient was worked up for suspected DVT and underwent an ultrasound venogram. This ruled out a DVT but identified a left SFA PSA. The patient was referred to the vascular and orthopaedic surgery services. Subsequently, the patient underwent a computed tomography (CT) lower limb angiogram which further delineated the left SFA PSA as 2.7cm in diameter. 

**Figure 1:**
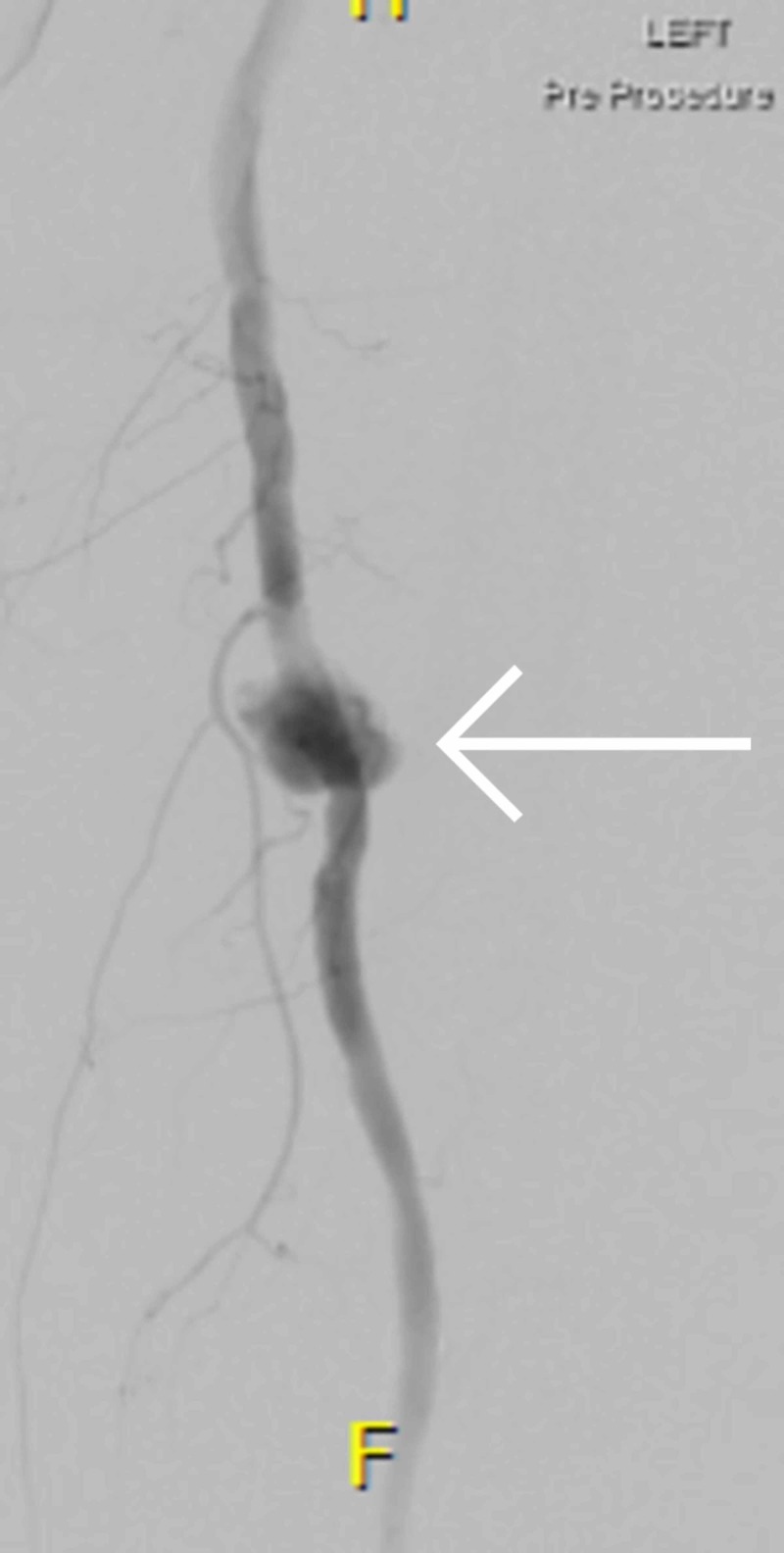
Angiogram demonstrating the left SFA PSA pre-operatively.

**Figure 2:**
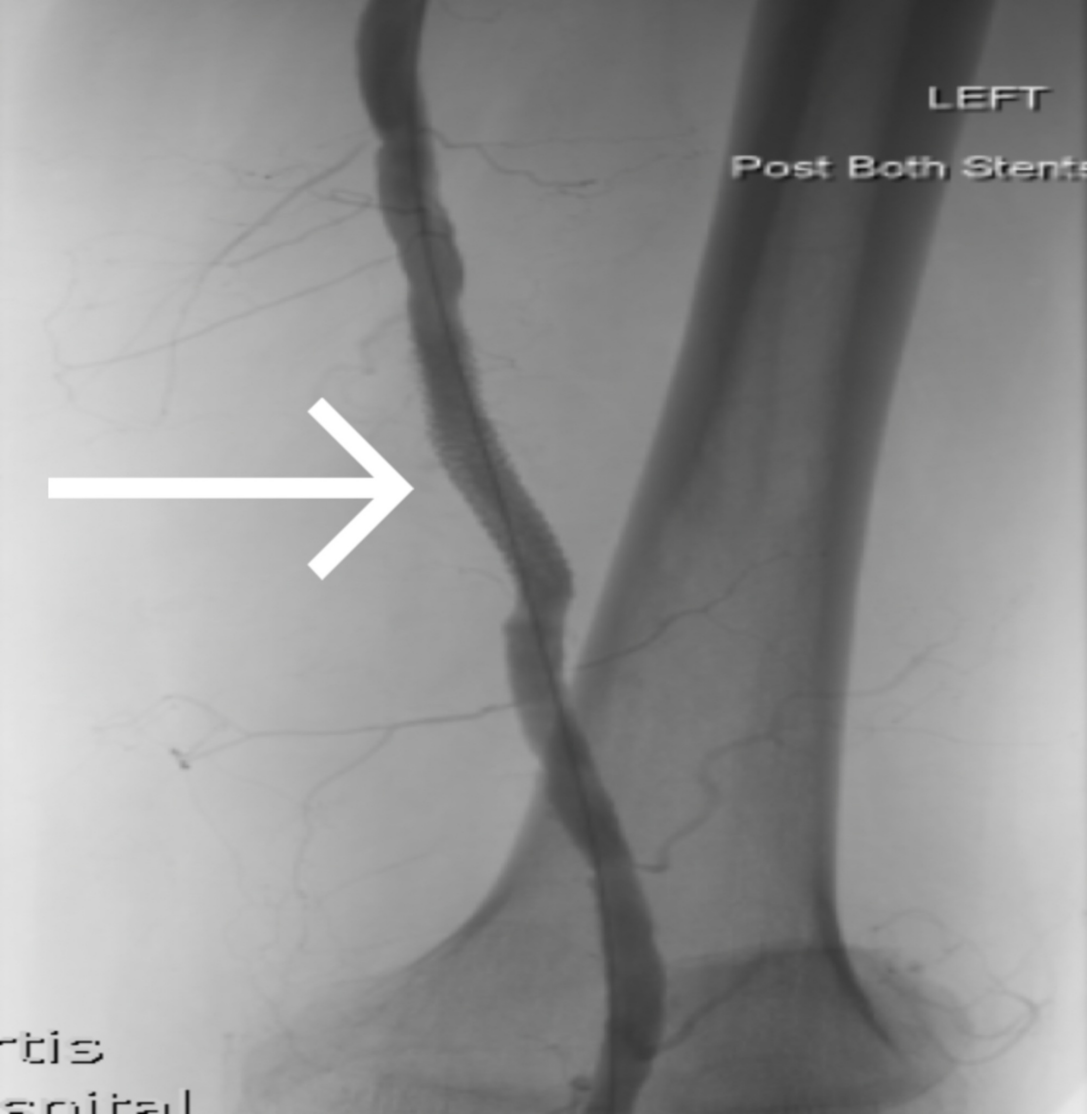
Intraoperative fluoroscopy demonstrating the left SFA PSA after covered stenting post-operatively

The patient underwent covered stenting under the vascular surgery service of the left SFA. Post operatively, the patient did extremely well. The swelling resolved in the patient’s thigh and the pain went away. The patient continues to have knee pain and due to severe osteoarthritis, the patient may end up undergoing a TKR. If the patient does undergo a TKR, it will be performed without the use of a tourniquet and he will continue to take the oral anticoagulant perioperatively. Figure 1 demonstrates the left SFA PSA pre-operatively. Figure 2 demonstrates the left SFA PSA after covered stenting post-operatively.

A PSA of the left SFA after knee arthroscopy has not been documented in the literature and we propose that this is the first PSA of the left SFA after knee arthroscopy to be documented. Of note, there have been numerous PSA in various anatomical locations reported previously after knee arthroscopy but none described in the SFA [3]. 

## Discussion

This case raises many discussion points. If a patient has thigh pain and swelling post knee arthroscopy, it is important to rule out a DVT. Although it has a low incidence of 0.4% after knee arthroscopy, the increased incidence relative to the general population is 14-fold higher [4]. 

Over 47 cases of PSA after knee arthroscopy have been reported [3]. None of these cases occurred in the SFA. A PSA is defined as a pulsating, encapsulated haematoma in communication with the lumen of a ruptured vessel [5]. Although these injuries are rarely associated with knee arthroscopy, when they occur, they are associated with direct vessel injury when operating near the knee joint as the popliteal vessels are in close proximity to the joint capsule. It is suggested that an injury can occur secondary to pressure to the vessels around the knee [3]. 

The etiology of the PSA in this case is unknown. It definitely is not from direct injury during the knee arthroscopy. As the PSA occurred in the thigh directly under the tourniquet, we believe it occurred as a result of the tourniquet. The tourniquet was inflated to 300 mmHg. This is a routine pressure used for knee arthroscopic procedures. The pressure from the tourniquet may have inflicted an indirect injury to the SFA. We were unable to find any PSA in the literature related to tourniquet usage. 

Although it is likely the tourniquet caused the PSA, the patient was also taking Apixaban which may have contributed to the formation of it. Apixaban is an oral anticoagulant and works by directly inhibiting both free and clot bound factor Xa, in turn preventing thrombin formation and further clotting [6]. There is only one case report which associates Apixaban with spontaneous formation of a PSA [6]. Apixaban has been found to cause a PSA in the setting of procedural complications but this is extremely rare [6]. Therefore, should Apixaban be held peri-operatively for high risk elective knee arthroscopies in the future? The summary of product characteristics for Apixaban recommends that Apixaban be discontinued 24 - 48 hours pre-operatively depending on the risk of bleeding intra-operatively [7]. 

One could question the merits of performing a knee arthroscopy in an elderly osteoarthritic gentleman. This patient stated that he did not want a knee replacement and had many medical co-morbidies. He had multiple injections which no longer provided benefit. In consultation with the patient, shared decision making was made regarding the potential benefit of undergoing knee arthroscopy rather than a TKR. It was decided to proceed with the knee arthroscopy. 

This patient is a vasculopath and whether this made him more susceptible to a PSA is unclear but likely. We recommend that when consenting a patient for a knee arthroscopy, it is important to include damage to vascular structures which can lead to the development of a PSA as part of the consent process. We suggest attempting the knee arthroscopy without an inflated tourniquet if at all possible. There is good evidence to suggest that dilute epinephrine saline irrigation can decrease the need for tourniquet use [8].

This patient has done well after treatment of this complication. This case highlights the importance of getting the surgical indication correct and investigating a patient thoroughly who presents with unexplained post operative swelling and pain. One should have a low threshold to investigate for a DVT and a PSA should be part of the differential diagnosis. 

## Conclusions

This case highlights the importance of investigating a patient thoroughly who presents with unexplained post operative swelling and pain. One should have a low threshold to investigate for a DVT and a PSA should be part of the differential diagnosis. 
